# Interactions with Commensal and Pathogenic Bacteria Induce HIV-1 Latency in Macrophages through Altered Transcription Factor Recruitment to the Long Terminal Repeat

**DOI:** 10.1128/JVI.02141-20

**Published:** 2021-03-10

**Authors:** Gregory A. Viglianti, Vicente Planelles, Timothy M. Hanley

**Affiliations:** a Department of Microbiology, Boston University School of Medicine, Boston, Massachusetts, USA; b Department of Pathology, University of Utah Health, Salt Lake City, Utah, USA; Emory University

**Keywords:** *Escherichia coli*, HIV-1, *Neisseria gonorrhoeae*, interferon regulatory factors, latency, macrophages, Toll-like receptors

## Abstract

Macrophages are infected by human immunodeficiency virus type 1 (HIV-1) *in vivo* and contribute to both viral spread and pathogenesis. Recent human and animal studies suggest that HIV-1-infected macrophages serve as a reservoir that contributes to HIV-1 persistence during antiretroviral therapy. The ability of macrophages to serve as persistent viral reservoirs is likely influenced by the local tissue microenvironment, including interactions with pathogenic and commensal microbes. Here, we show that the sexually transmitted pathogen Neisseria gonorrhoeae (gonococcus [GC]) and the gut-associated microbe Escherichia coli, which encode ligands for both Toll-like receptor 2 (TLR2) and TLR4, repressed HIV-1 replication in macrophages and thereby induced a state reminiscent of viral latency. This repression was mediated by signaling through TLR4 and the adaptor protein Toll/interleukin 1 (IL-1) receptor domain-containing adapter-inducing beta interferon (IFN) (TRIF) and was associated with increased production of type I interferons. Inhibiting TLR4 signaling, blocking type 1 interferon, or knocking down TRIF reversed lipopolysaccharide (LPS)- and gonococcus-mediated repression of HIV-1. Finally, the repression of HIV-1 in macrophages was associated with the recruitment of interferon regulatory factor 8 (IRF8) to the interferon-stimulated response element (ISRE) downstream of the HIV-1 5′ long terminal repeat (LTR). Our data indicate that IRF8 is responsible for repression of HIV-1 replication in macrophages in response to TRIF-dependent signaling during N. gonorrhoeae and E. coli coinfection. These findings highlight the potential role of macrophages as HIV-1 reservoirs, as well as the roles of the tissue microenvironment and coinfections as modulators of HIV-1 persistence.

**IMPORTANCE** The major barrier toward the eradication of HIV-1 infection is the presence of a small reservoir of latently infected cells, which include CD4^+^ T cells and macrophages that escape immune-mediated clearance and the effects of antiretroviral therapy. There remain crucial gaps in our understanding of the molecular mechanisms that lead to transcriptionally silent or latent HIV-1 infection of macrophages. The significance of our research is in identifying microenvironmental factors, such as commensal and pathogenic microbes, that can contribute to the establishment and maintenance of latent HIV-1 infection in macrophages. It is hoped that identifying key processes contributing to HIV-1 persistence in macrophages may ultimately lead to novel therapeutics to eliminate latent HIV-1 reservoirs *in vivo*.

## Abstract (precis)

The major barrier toward the eradication of HIV-1 infection is the presence of a small reservoir of latently infected cells, which include CD4^+^ T cells and macrophages that escape immune-mediated clearance and the effects of antiretroviral therapy. There remain crucial gaps in our understanding of the molecular mechanisms that lead to transcriptionally silent or latent HIV-1 infection of macrophages.

## INTRODUCTION

Macrophages are among the immune cells located within the gastrointestinal and genitourinary mucosae thought to play a role in human immunodeficiency virus type 1 (HIV-1) sexual transmission and pathogenesis (1–3). A number of studies examining either HIV-1 infection of human vaginocervical or gastrointestinal tissue explants or rhesus macaque simian immunodeficiency virus (SIV_mac_) infection in rhesus macaque animal models have shown that macrophages are among the first cells infected during mucosal transmission (2, 4, 5). Macrophages can be productively infected with HIV-1 and are thought to be a source of virus persistence *in vivo* (6). Given their role in transmission, pathogenesis, and viral persistence, it is important to understand how the local mucosal microenvironment and cellular signaling pathways modulate interactions between macrophages and HIV-1.

Sexually transmitted infections (STIs) have been shown to be cofactors that enhance HIV-1 transmission (7). Neisseria gonorrhoeae (gonococcus [GC]) is a nonulcerative STI that is thought to augment mucosal transmission of HIV-1, both by inducing inflammation and by directly activating virus infection and replication (8–13). The role of GC in HIV-1 persistence is less well understood. Several studies have implicated GC-encoded pathogen-associated molecular patterns (PAMPs) as mediators of both inflammation and HIV-1 activation in target cells such as macrophages; however, the interactions between GC and macrophages are complex. GC encodes PAMPs capable of engaging Toll-like receptors (TLRs), including TLR2, TLR4, and TLR9 (14, 15). While the effects of coinfection with live GC on HIV-1 replication in macrophages have not been reported, purified lipooligosaccharide (LOS), as well as Escherichia coli lipopolysaccharide (LPS), have been shown to repress virus replication through the production of type 1 interferons (IFNs) (16, 17). In the case of LPS, repression is due to undefined effects at the level of gene expression. Although it is not entirely clear how TLR2 signaling affects HIV-1 expression in macrophages, studies have shown that purified TLR2 ligands activate virus replication in macrophages (18) and in latently infected T cells (19).

Here, we demonstrate that coinfection with GC and E. coli repress HIV-1 expression in macrophages. To investigate the underlying mechanism(s) responsible for this repression, we examined the individual effects of TLR2 and TLR4 signaling on HIV-1 expression in macrophages. TLR2 signaling activated HIV-1 expression in macrophages, whereas TLR4 signaling repressed virus expression. Importantly, TLR4 signaling overcame the activation effects of TLR2 signaling in macrophages. The TLR4-mediated repression of HIV-1 in macrophages coinfected with GC or E. coli was dependent on signaling through Toll/interleukin 1 (IL-1) receptor domain-containing adapter-inducing IFN-β (TRIF) and required type 1 IFN production. Finally, we showed that TLR4 signaling leads to the late-phase recruitment of IRF8 to the interferon-stimulated response element (ISRE) downstream of the HIV-1 5′ LTR in infected macrophages. Taken together, our data suggest that TRIF-mediated signaling represses HIV-1 replication in response to GC or E. coli coinfection in an IRF8-dependent manner and shifts macrophages from a state of robust HIV-1 expression to a state of persistent low-level/latent infection.

## RESULTS

### HIV-1 gene expression in MDMs is enhanced or repressed in a TLR-specific manner.

**FIG 1:**
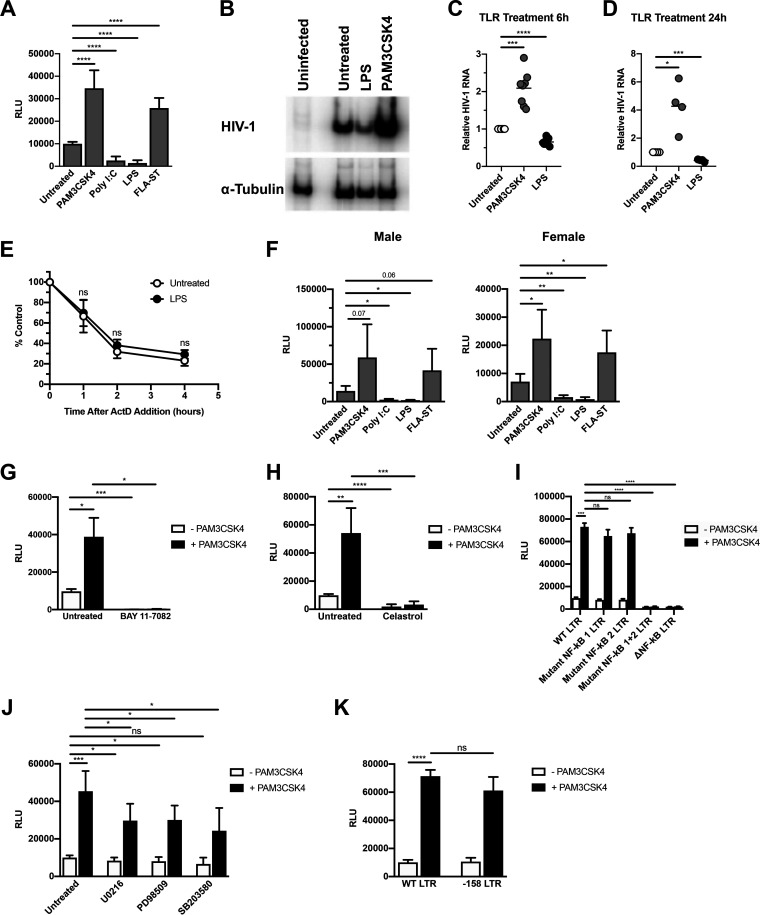
Treatment with purified Toll-like receptor (TLR) ligands alters HIV-1 replication at the level of transcription. (A) Monocyte-derived macrophages (MDMs) were infected with a single-round, replication-defective HIV-luciferase reporter virus and, 48 h after infection, were treated with the TLR2 ligand PAM3CSK4 (100 ng/ml), the TLR3 ligand poly(I·C) (25 μg/ml), the TLR4 ligand lipopolysaccharide (LPS) (100 ng/ml), or the TLR5 ligand FLA-ST (100 ng/ml) for 18 h. The cells were then lysed and assayed for luciferase activity. Bars represent the mean (± standard deviation [SD]) of 11 donors, each donor tested in triplicate. (B to D) MDMs were infected as described above. At 48 h after infection, cells were treated with PAM3CSK4 (100 ng/ml) or LPS (100 ng/ml) for 6 h (B and C) or 24 h (D). Cells were then lysed and assayed for viral RNA accumulation by reverse transcription-PCR (RT-PCR). Shown are data from one representative donor at 6 h (B) and composite data from eight donors at 6 h (C) and four donors at 24 h (D). (E) MDMs were infected as in panel A. At 48 h after infection, cells were treated with the TLR4 ligand LPS (100 ng/ml) for 4 h. Cells were then treated with actinomycin D (10 μg/ml) to inhibit transcription. Total cytoplasmic RNA was prepared from the treated cultures at the indicated time points following actinomycin D treatment and analyzed by reverse transcription-quantitative PCR (RT-qPCR) for the expression of HIV-1 RNA. The data are the means (± SD) from four donors. (F) MDMs were infected and treated with TLR ligands as in (A). The cells were then lysed and assayed for luciferase activity. Bars represent the mean (± SD) of five male donors and five female donors; each donor was tested in triplicate. Although virus replication was decreased overall in MDMs from female donors compared to MDMs from male donors, it was activated by treatment with PAM3CSK4 and FLA-ST and repressed by poly(I·C) and LPS, in a manner similar to that seen in MDMs from male donors. (G to H) MDMs were infected as in panel A, and, 48 h after infection, cells were treated with PAM3CSK4 (100 ng/ml) in the presence or absence of 10 μM BAY 11-7082 (G) or 10 μM celastrol (H) for 18 h. The cells were then lysed and assayed for luciferase activity. The data are the mean (± SD) of three donors (BAY 11-7082) or six donors (celastrol); each donor was tested in triplicate. (I) HEK293-TLR2^CFP^TLR1^YFP^ cells were transfected with HIV-1 long terminal repeat (LTR)-luciferase reporter constructs with intact NF-κB, mutated NF-κB, or deleted NF-κB binding sites. Following transfection, cells were treated with PAM3CSK4 (100 ng/ml) for 18 h and then harvested and assayed for luciferase activity. Data represent the mean (± SD) of three independent experiments, each performed in triplicate. (J) MDMs were infected as in (A), and 48 h after infection, cells were treated with PAM3CSK4 (100 ng/ml) in the presence or absence of U0126 (10 μM), PD98059 (50 μM), or SB203580 (10 μM) for 18 h. Cells were then lysed and assayed for luciferase activity. The data are the mean (± SD) of six donors; each donor was tested in triplicate. (K) HEK293-TLR2^CFP^TLR1^YFP^ cells were transfected with HIV-1 LTR-luciferase reporter constructs with intact AP-1 sites (wild-type [WT] LTR) or deleted AP-1 binding sites (−158 LTR). Following transfection, cells were treated with PAM3CSK4 (100 ng/ml) for 18 h and then harvested and assayed for luciferase activity. Data are the mean (± SD) of three independent experiments, each performed in triplicate. *, *P* < 0.05; **, *P* < 0.01; ***, *P* < 0.001; ****, *P* < 0.0001; ns, not significant.

To determine how purified TLR ligands affected HIV-1 gene expression, monocyte-derived macrophages (MDMs) were infected with a single-round infectious HIV-1 reporter virus, and then treated with ligands for TLR2, TLR3, TLR4, or TLR5. Ligands that activated TLR2 or TLR5 enhanced HIV-1 replication, whereas ligands for TLR3 or TLR4 repressed HIV-1 expression (Fig. 1A). The effects of TLR ligands on HIV-1 replication occurred at the level of transcription, as treatment with the TLR2/1 ligand PAM3CSK4 led to an increase in HIV-1 mRNA accumulation, whereas treatment with the TLR4 ligand LPS led to a decrease in HIV-1 transcript levels (Fig. 1B to D). TLR treatment had no effect on viral RNA stability, as viral RNA from LPS-treated MDMs had a similar decay rate to that from untreated MDMs (Fig. 1E). Recent studies have demonstrated that myeloid cells from males and females have different susceptibilities to HIV-1 infection, largely due to differential levels of innate immune responses and steroid hormones (20–22). We therefore sought to determine whether there was a sex-based difference in the response to TLR ligand treatment in MDMs. We found that TLR stimulation had similar effects on HIV-1 expression in MDMs from both male and female donors (Fig. 1F). These results indicate that MyD88-dependent signaling enhances HIV-1 transcription, whereas TRIF-dependent signaling inhibits HIV-1 transcription in MDMs.

MyD88-dependent TLR signaling leads to the activation of both NF-κB and AP-1 transcription factors, among others (23). The HIV-1 LTR contains binding sites for both NF-κB and AP-1. The two NF-κB sites are thought to be essential for HIV-1 transcription (24, 25), whereas the AP-1 sites, while not essential, are thought to enhance HIV-1 transcription (26, 27). Previous studies demonstrated that treatment of HIV-infected MDMs with the TLR2/TLR1 ligand PAM3CSK4 led to an increased association of the p65 subunit of NF-κB and the c-*fos* subunit of AP-1 with the 5′ LTR, which in turn correlated with increased virus replication (18); however, the contributions made by each pathway to TLR2-mediated activation have not been previously characterized. To determine the roles of NF-κB and AP-1 in TLR2-activated HIV replication in MDMs, HIV-1- infected cells were treated with either BAY 11-7082, an inhibitor of IκB kinase (28); celastrol, a small-molecule inhibitor of the IκB kinase complex (29); or inhibitors that disrupt AP-1 signaling. As shown in Fig. 1G and H, BAY 11-7082 and celastrol treatment completely ablated TLR2/1-enhanced HIV-1 expression. Similarly, the use of an LTR-based reporter construct with mutations in the NF-κB binding sites did not result in increased gene expression in response to TLR2 signaling (Fig. 1I). Treatment of HIV-infected macrophages with inhibitors of kinases upstream of AP-1 activation, such as MEK1/2 (U0126, PD98509), and p38 (SB203580), resulted in modest, but reproducible, decreases in TLR2-mediated activation of HIV-1 (Fig. 1J). Similarly, LTR reporter constructs lacking AP-1 binding sites were activated in response to TLR2 signaling at levels similar to that of the wild-type (WT) construct, further demonstrating the nonessential role of AP-1 in TLR2-mediated HIV-1 activation (Fig. 1K). Although the regulation of HIV-1 transcription through multiple transcription factor binding sites in and adjacent to the 5′ LTR is complex, these data suggest that, in MDMs, TLR2-activated HIV-1 expression is mediated primarily through NF-κB, with a minor contribution from AP-1 signaling.

### Coinfection with Neisseria gonorrhoeae or Escherichia coli represses HIV-1 replication in MDMs.

**FIG 2:**
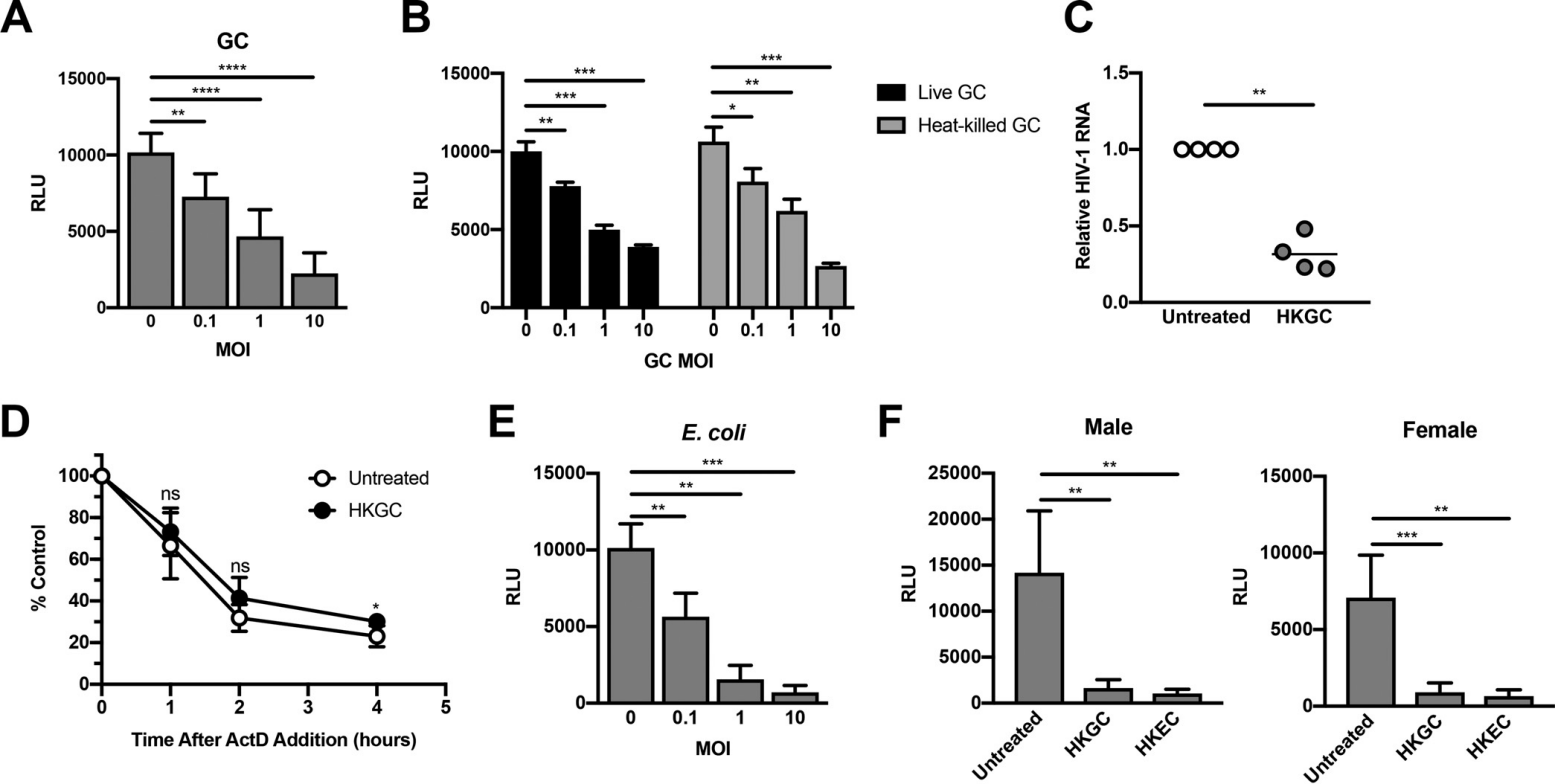
Treatment with intact Neisseria gonorrhoeae and Escherichia coli represses HIV-1 replication at the level of transcription. (A) MDMs were infected with a single-round, replication-defective HIV-luciferase reporter virus and, 48 h after infection, were cultured overnight with increasing amounts of gonococcus (GC). Cells were then lysed and assayed for luciferase activity. Bars represent mean (± SD) of seven donors; each donor was tested in triplicate. (B) MDMs were infected as in panel A and, 48 h after infection, were cultured with increasing amounts of live or heat-killed (56°C treatment) GC overnight. The cells were then lysed, and luciferase activity was measured. The data are the mean (± SD) of three donors; each donor was tested in triplicate. (C) MDMs were infected as described above and, 48 h after infection, were treated with heat-killed GC (HKGC) at a multiplicity of infection (MOI) of 10 for 24 h. Cells were then lysed and assayed for viral RNA accumulation by RT-qPCR. Shown are data from four donors. (D) MDMs were infected as in panel A and, 48 h after infection, cells were treated with heat-killed GC (MOI = 10) for 4 h. Cells were then treated with actinomycin D (10 μg/ml) to inhibit transcription. Total cytoplasmic RNA was prepared from the treated cultures at the indicated time points following actinomycin D treatment and analyzed by RT-qPCR for the expression of HIV-1 RNA. The data are the means (± SD) from four donors. (E) MDMs were infected as in panel A and, 48 h after infection, the cells were cultured overnight with increasing amounts of E. coli. Cells were then lysed and assayed for luciferase activity. Bars represent mean (± SD) of four donors, with each donor tested in triplicate. (F) MDMs were infected as in panel A and, 48 h after infection, cells were treated with heat-killed GC (MOI = 10) or heat-killed E. coli (HKEC) (MOI = 10) for 18 h. The cells were then lysed and assayed for luciferase activity. Bars represent the mean (± SD) of five male donors and five female donors; each donor was tested in triplicate. Although virus replication was decreased overall in MDMs from female donors compared to that in MDMs from male donors, it was repressed by HKGC and HKEC in a manner similar to that seen in MDMs from male donors. *, *P* < 0.05; **, *P* < 0.01; ***, *P* < 0.001; ****, *P* < 0.0001; ns, not significant.

Our preliminary studies using purified TLR ligands in isolation suggested that different TLR signaling cascades had diverse effects on HIV-1 replication. Since most pathogens encode multiple TLR ligands, we sought to determine the effects of intact pathogens on HIV-1 replication. We incubated HIV-infected MDMs with N. gonorrhoeae (gonococcus [GC]), which expresses ligands for TLR2, TLR4, and TLR9. We found that increasing amounts of GC led to a dose-dependent decrease in HIV-1 replication in MDMs (Fig. 2A). Bacterial replication was not required for these effects, as heat-killed GC led to repression of HIV-1 replication in MDMs (Fig. 2B). GC-mediated repression occurred at the level of viral transcription (Fig. 2C) and did not decrease viral RNA stability (Fig. 2D). In addition, repression of HIV-1 replication is not specific for GC, but may be a generalized response to Gram-negative bacteria, as coinfection with E. coli also repressed HIV-1 replication in MDMs in a manner similar to GC (Fig. 2E). Similar to what we observed with purified LPS, the biological sex of the donors had no effect on GC- or E. coli-mediated HIV-1 repression in MDMs (Fig. 2F). Despite the presence of both activating (TLR2) and repressing (TLR4) TLR ligands, both GC and E. coli mediated repression of HIV-1 replication in macrophages. This finding raised several possibilities, as follows: (i) the dominance of TLR4 signaling over TLR2 signaling in MDMs; (ii) different expression levels of TLR2-, TLR4-, and TLR4-associated molecules such as CD14 and MD-2 on MDMs; (iii) different cytokine profiles produced in response to GC or E. coli; and/or (iv) variable expression of signaling molecules downstream of TLRs. These scenarios were further explored.

### TLR4 signaling is dominant in MDMs.

**FIG 3:**
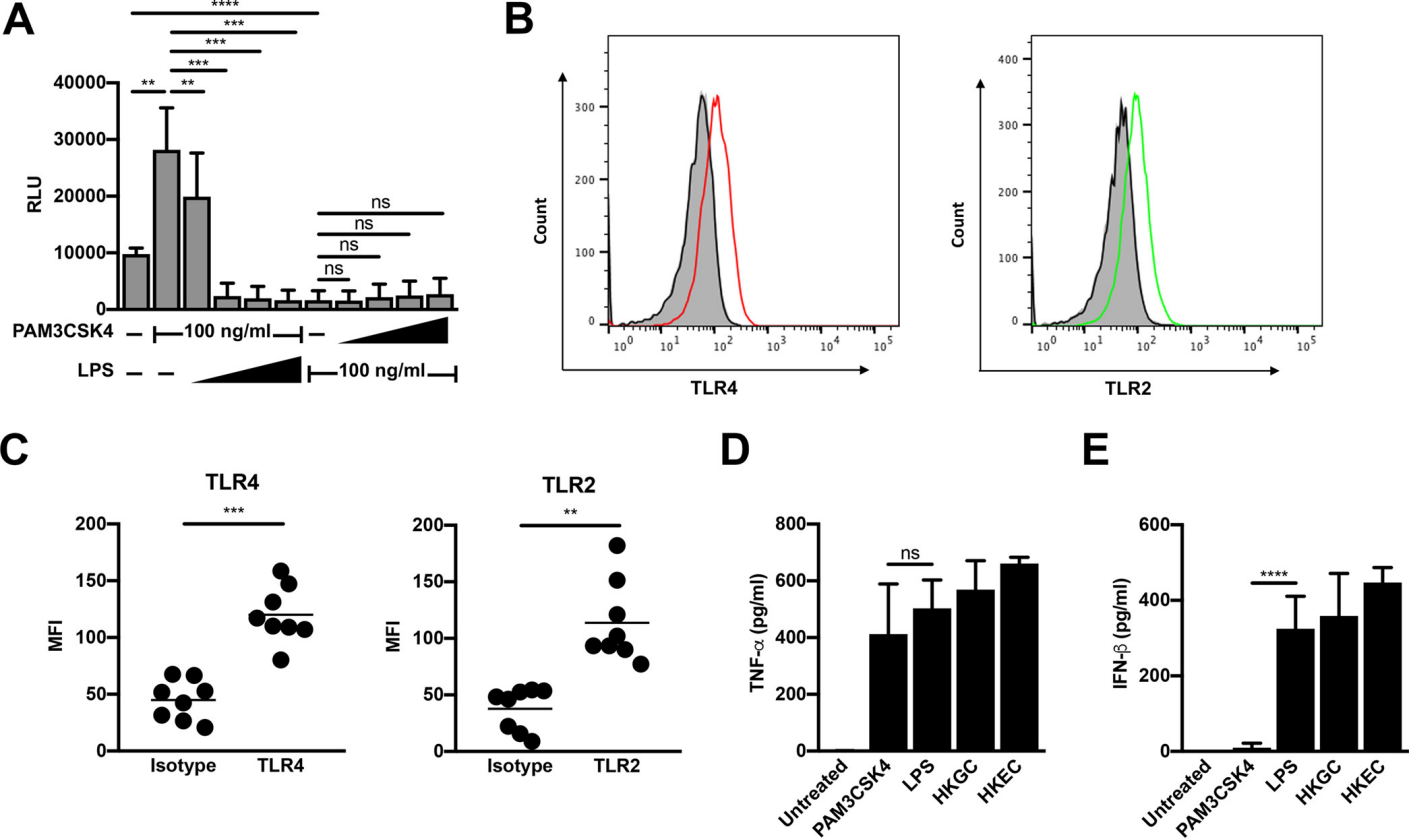
TLR4 signaling is dominant in MDMs. (A) MDMs were infected with a single-round, replication-defective HIV-luciferase reporter virus and, 48 h after infection, were treated with a fixed concentration of PAM3CSK4 (100 ng/ml) and increasing concentrations of LPS (1 to 1,000 ng/ml, as indicated) or a fixed concentration of LPS (100 ng/ml) and increasing concentrations of PAM3CSK4 (1 to 1,000 ng/ml, as indicated) for 18 h. Cells were then lysed and assayed for luciferase activity. The data are the mean (± SD) of six donors; each donor was tested in triplicate. (B and C) At 8 days postisolation, MDMs were stained with antibodies against TLR2 or TLR4 or relevant isotype controls. Receptor expression was assessed by flow cytometry. Histograms from one representative donor are shown in panel B. Gray, unstained cells; black line, isotype control; red line, TLR4; green line, TLR2. Mean fluorescent intensity (MFI) ± SD from eight donors is depicted in panel C. (D and E) MDMs were treated with the TLR2 ligand PAM3CSK4 (100 ng/ml), the TLR4 ligand LPS (100 ng/ml), heat-killed GC (MOI = 10), or heat-killed E. coli (MOI = 10) for 18 h. Cell supernatant was harvested, filtered through a 0.2-μm filter, and analyzed by enzyme-limited immunosorbent assay (ELISA) for tumor necrosis factor alpha (TNF-α) (D) and beta interferon (IFN-β) (E) production. Data represent mean (± SD) of seven donors (four donors for heat-killed E. coli). *, *P* < 0.05; **, *P* < 0.01; ***, *P* < 0.001; ****, *P* < 0.0001; ns, not significant.

To determine whether certain TLR pathways are dominant in MDMs, we performed cotreatments of HIV-infected MDMs with the TLR2 ligand PAM3CSK4 and the TLR4 ligand LPS. We found that increasing the concentration of LPS against a fixed concentration of PAM3CSK4 led to a reversal of TLR2-mediated activation of HIV-1 and, eventually, to repression of HIV-1 replication (Fig. 3A). Conversely, increasing the concentration of PAM3CSK4 against a fixed concentration of LPS did not reverse LPS-mediated repression of HIV-1 (Fig. 3A). Flow cytometry was used to determine that the different responses of MDMs were likely not due to receptor expression, as MDMs express both TLR2 and TLR4 (Fig. 3B and C). In addition, MDMs produced both tumor necrosis factor alpha (TNF-α) and beta interferon (IFN-β) in response to LPS treatment, GC coinfection, and E. coli coinfection. Whereas treatment of MDMs with LPS resulted in a similar cytokine profile to that of coinfection, treatment of MDMs with the TLR2/1-ligand PAM3CSK4 resulted in the production of TNF-α, but not in appreciable levels of IFN-β (Fig. 3D and E). Taken together, our data suggest that TLR4 signaling, which negatively regulates LTR-driven gene expression, is dominant in MDMs.

### LPS- and GC-mediated repression of HIV-1 in MDMs is dependent on TRIF-mediated type I IFN production.

**FIG 4:**
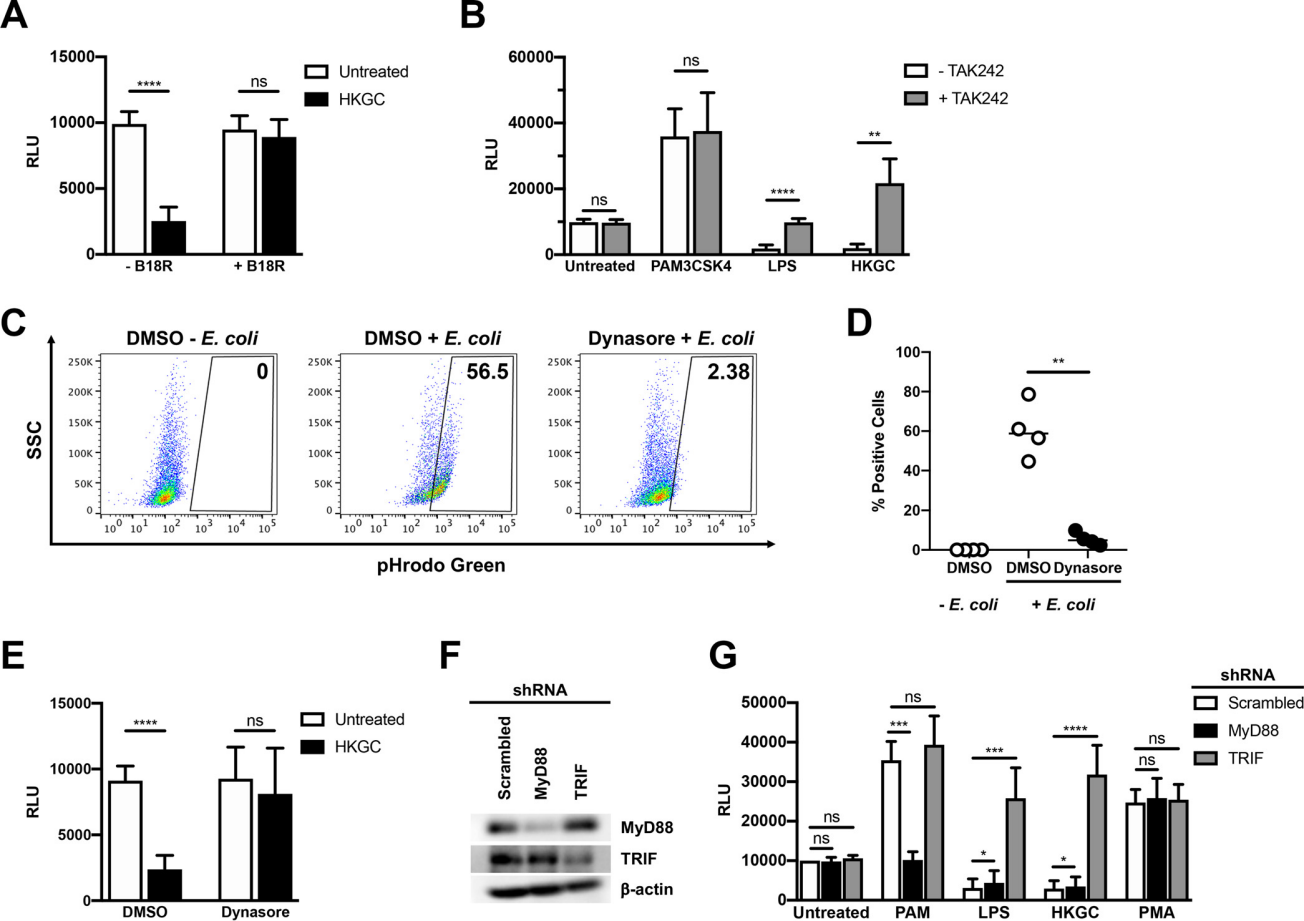
LPS- and GC-mediated repression of HIV-1 replication in MDMs requires TLR4, TRIF, and type I IFNs. (A) MDMs were infected with a single-round, replication-defective HIV-luciferase reporter virus and, 48 h after infection, cells were treated with GC (MOI = 10) in the absence (white bars) or presence (black bars) of B18R (100 ng/ml) for 18 h. The cells were then lysed and assayed for luciferase activity. The data are the mean (± SD) of seven donors; each donor was tested in triplicate. (B) MDMs were infected as in panel A and, 48 h after infection, were treated with PAM3CSK4 (100 ng/ml), LPS (100 ng/ml), or heat-killed GC (MOI = 10) in the absence (white bars) or presence (gray bars) of TAK242 (1 μg/ml) for 18 h. The cells were then lysed and assayed for luciferase activity. The data are the mean (± SD) of six donors; each donor was tested in triplicate. (C and D) MDMs were incubated with dimethyl sulfoxide (DMSO) or the dynamin inhibitor Dynasore (80 μM) for 15 min at 37°C. The cells were washed with phosphate-buffered saline (PBS) and incubated with pHrodo green E. coli (1 mg/ml) for 2 h at 37°C. Endocytosis/phagocytosis was measured by flow cytometry. Shown are data from one representative donor (C) and composite data from four donors (D). (E) MDMs were infected as in panel A and, 48 h after infection, were treated with vehicle control (white bars) or with Dynasore (80 μM); black bars for 15 min prior to treatment with heat-killed GC (MOI = 10) for 18 h. The cells were then lysed and assayed for luciferase activity. The data are the mean (± SD) of six donors, each donor tested in triplicate. (F and G) MDMs were transfected with a control scrambled shRNA, shRNA targeting MyD88, or shRNA targeting TRIF. Knockdown of protein expression was detected by Western blot analysis (F). Transfected MDMs were infected with a single-round, replication-defective HIV-luciferase reporter virus and, 48 h after infection, were treated with PAM3CSK4 (100 ng/ml), LPS (100 ng/ml), heat-killed GC (MOI = 10), or phorbol myristate acetate (PMA) (10 nM) for 18 h. The cells were then lysed and assayed for luciferase activity (G). The data are the mean (± SD) of six donors. *, *P* < 0.05; **, *P* < 0.01; ***, *P* < 0.001; ****, *P* < 0.0001; ns, not significant.

Since LPS and GC both induce type I IFN production, whereas the TLR2 ligand PAM3CSK4 does not, we wished to determine whether GC-stimulated production of IFN-α/β contributes to repression of HIV-1 in MDMs. We found that treatment of HIV-infected MDMs with the vaccinia virus-encoded soluble type I IFN receptor B18R reversed GC-mediated inhibition of HIV-1 replication, suggesting that TLR4-mediated IFN production is required for HIV-1 repression by GC (Fig. 4A). Since both purified TLR4 ligands and GC, which encodes ligands for TLR2, TLR4, and TLR9, repress HIV-1 replication in MDMs, we predicted that downstream effector molecules of TLR4 signaling would contribute to the repression of HIV-1 replication in MDMs. First, we confirmed that TLR4 signaling was responsible for GC-mediated HIV-1 repression in MDMs. Treatment with the TLR4-specific inhibitor TAK242 reversed the LPS- and GC-dependent repression of HIV-1 in MDMs (Fig. 4B). Treatment with TAK242 had no effect on TLR2-mediated activation of HIV-1 replication in MDMs, consistent with reports that TAK242 is specific for TLR4 (30).

It has been shown that TLR4, which can utilize both MyD88 and TRIF adaptor proteins, initiates different signaling pathways dependent upon its cellular location. Cell-surface TLR4 engagement leads to MyD88-dependent signaling, whereas endosomal TLR4 engagement leads to TRIF-dependent signaling (31). To examine whether TRIF-dependent signaling is responsible for HIV-1 repression, we blocked dynamin-dependent endocytosis of TLR4 with Dynasore, which prevents TRIF-dependent signaling while leaving MyD88-dependent signaling intact. As shown, blocking endocytosis-mediated TLR4 internalization (Fig. 4C and D) reversed GC-mediated repression of HIV-1 in MDMs (Fig. 4E). Given the ability of GC to signal through both TLR2-MyD88 and TLR4-TRIF, one might expect the inhibition of type I IFN signaling by B18R or the inhibition of endocytosis by Dynasore to lead to augmented viral gene expression through intact MyD88 signaling. However, we did not observe this, likely due to incomplete inhibition of either IFN signaling or endocytosis.

To confirm the role of MyD88 in TLR2-mediated HIV-1 activation and TRIF in TLR4-mediated HIV-1 repression, we used short hairpin RNAs (shRNAs) to knock down the two molecules in HIV-infected MDMs (Fig. 4F). Knockdown of MyD88 led to a loss of TLR2-mediated HIV-1 activation but had no effect on LPS- or GC-mediated HIV-1 repression (Fig. 4G). In contrast, knockdown of TRIF had no effect on TLR2-mediated HIV-1 activation, but reversed LPS- and GC-mediated repression of HIV-1 replication (Fig. 4G). Knockdown of either MyD88 or TRIF had no effect on the activation of HIV-1 by the phorbol ester phorbol myristate acetate (PMA), which signals directly through protein kinase C, independently of TLRs (Fig. 4G). These data suggest that the TLR4-TRIF-type I IFN axis in MDMs leads to GC- and E. coli-mediated repression of HIV-1 replication.

### TLR4 signaling leads to differential IRF recruitment to the HIV-1 LTR.

**FIG 5:**
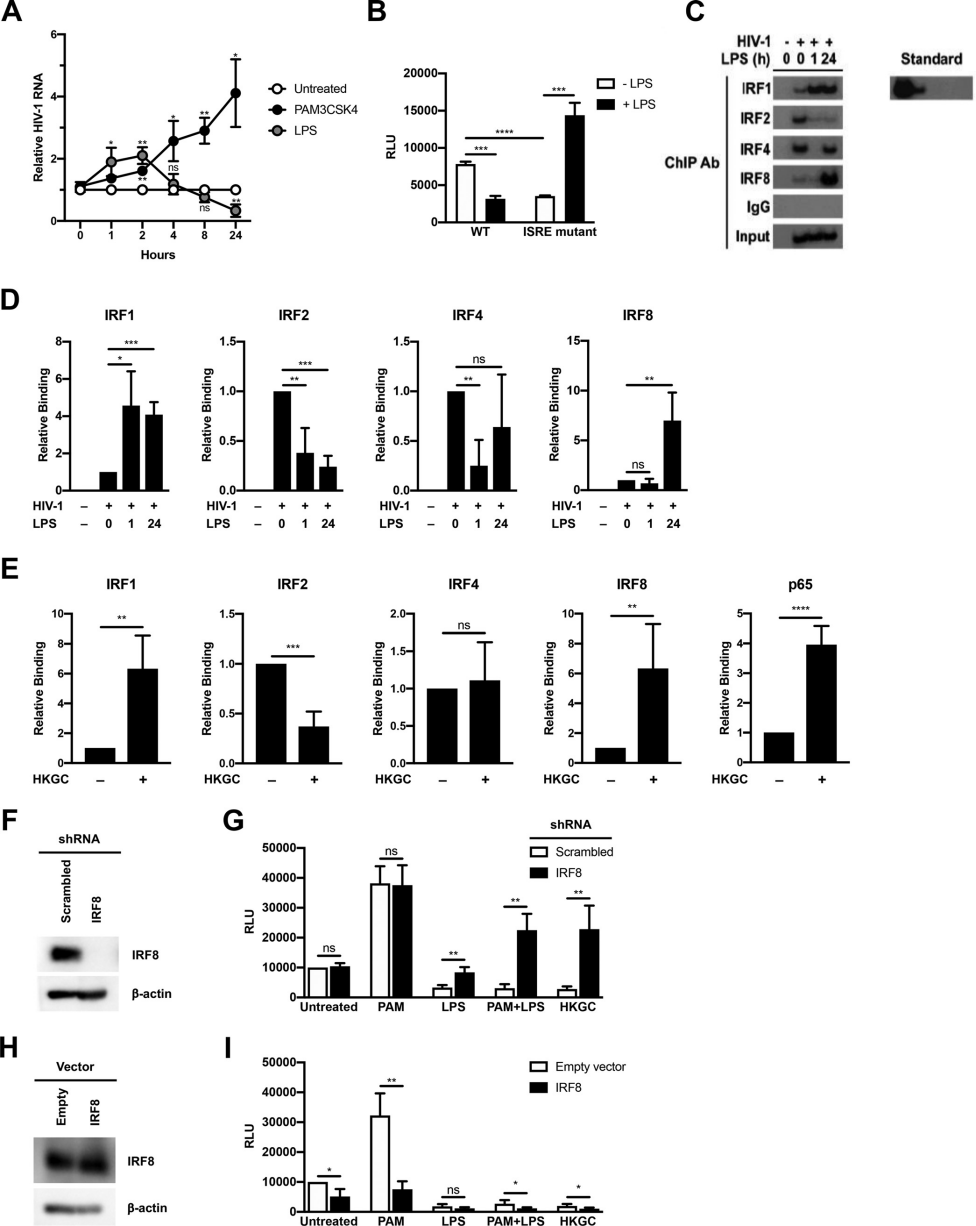
LPS- and GC-mediated repression of HIV-1 in MDMs is associated with changes in interferon regulatory factor (IRF) recruitment to the interferon-stimulated response element (ISRE). (A) MDMs were infected with a single-round, replication-defective HIV-luciferase reporter virus and, 48 h after infection, were treated with PAM3CSK4 (100 ng/ml) or LPS (100 ng/ml). At various time points after TLR stimulation, cells were harvested, lysed, and total cytoplasmic RNA was extracted. Viral RNA accumulation was assessed by RT-PCR. The data are the mean (± SD) of four donors. (B) HEK293-TLR4^CFP^/MD-2/CD14 cells were transfected with HIV-1 LTR/Gag-leader sequence (GLS)-luciferase reporter constructs with an intact ISRE or mutated ISRE binding site. Following transfection, cells were treated with LPS (100 ng/ml) for 18 h and then harvested and assayed for luciferase activity. Data are the mean (± SD) of three independent experiments, each performed in triplicate. (C and D) MDMs were infected with a single-round replication-defective HIV-GFP reporter virus and, 48 h after infection, cells were treated with LPS (100 ng/ml). At either 1 or 24 h after LPS treatment, cells were fixed with formaldehyde, lysed, sonicated, and subjected to immunoprecipitation with antibodies against IRF1, IRF2, IRF4, IRF8, or rabbit IgG (isotype control). Association with the HIV-1 ISRE was assessed by PCR using HIV-1 specific primers. Data from one representative donor (C). Composite data representing the mean (± SD) from five donors (D). (E) MDMs were infected as in panel C and, 48 h after infection, were treated with heat-killed GC (MOI = 10). At 24 hours after GC treatment, cells were fixed with formaldehyde, lysed, sonicated, and subjected to immunoprecipitation with antibodies against IRF1, IRF2, IRF4, IRF8, NF-κB p65, or rabbit IgG (isotype control). Association with the HIV-1 ISRE was assessed by PCR using HIV-1-specific primers. Composite data from five donors are shown. (F and G) MDMs transfected with a controlled scrambled shRNA (white bars) or with shRNA targeting IRF8 (black bars). Knockdown of protein expression was detected by Western blot (F). Transfected MDMs were infected with a single-round, replication-defective HIV-luciferase reporter virus and, 48 h after infection, were treated with PAM3CSK4 (100 ng/ml), LPS (100 ng/ml), a combination of PAM3CSK4 and LPS (each at 100 ng/ml), or GC (MOI = 10) for 18 h. Cells were then lysed and assayed for luciferase activity (G). The experiment was performed using cells from five different donors. (H and I) MDMs transfected with an empty vector (white bars) or a vector encoding IRF8 (black bars). IRF8 protein expression was detected by Western blot analysis (H). Transfected MDMs were infected with a single-round, replication-defective HIV-luciferase reporter virus and, 48 h after infection, were treated with PAM3CSK4 (100 ng/ml), LPS (100 ng/ml), a combination of PAM3CSK4 and LPS (each at 100 ng/ml), or GC (MOI = 10) for 18 h. Cells were then lysed and assayed for luciferase activity (I). The experiment was performed using cells from four different donors. *, *P* < 0.05; **, *P* < 0.01; ***, *P* < 0.001; ****, *P* < 0.0001; ns, not significant.

Since type I IFN production is critical for GC- and E. coli-mediated HIV-1 repression in MDMs, we examined the role of interferon-stimulated genes (ISGs) in HIV-1 regulation. Previous studies have shown that ISGs are temporally regulated in macrophages in response to innate immune sensors and type I IFN signaling (32, 33). To determine whether the repressive effects of LPS were due to early- or late-phase ISGs, HIV-1-infected MDMs were treated with the TLR2 ligand PAM3CSK4 or the TLR4 ligand LPS, and total cytoplasmic RNA was extracted at various times posttreatment. Treatment of HIV-infected MDMs with the TLR2 ligand PAM3CSK led to a continuous increase in HIV-1 RNA levels (Fig. 5A). In contrast, treatment of HIV-infected MDMs with the TLR4 ligand LPS led to an initial short-lived increase in HIV-1 RNA levels; however, levels steadily declined thereafter (Fig. 5A), indicating that HIV-1 transcription displays a biphasic response to TLR4 stimulation in MDMs. This suggests that late-phase proteins induced by type I IFNs are responsible for TLR4-mediated decreases in HIV-1 transcription. It is known that HIV-1 contains an interferon-stimulated response element (ISRE) in the Gag-leader sequence (GLS), immediately downstream from the 5′ LTR. Because type I IFN is required for LPS- and GC-mediated repression of HIV-1 in MDMs, we assessed the role of the ISRE in this process using transient-transfection assays with mutated LTR-reporter constructs in HEK293 cells expressing TLR4, MD-2, and CD14. We found that LPS treatment repressed LTR-driven reporter-gene expression in cells expressing WT ISRE elements, but not in cells transfected with an LTR-luciferase construct containing a mutated ISRE (Fig. 5B). This suggests that transcription factor engagement of the ISRE governs TLR4-mediated HIV-1 repression.

Previous studies have shown that IRF1 and IRF2 both bind to this ISRE *in vitro* and that IRF1 and IRF2 expression are associated with enhanced HIV-1 transcription (34). Two other IRFs, IRF4 and IRF8, are also expressed in macrophages (35) and have been shown to increase in response to type I IFN signaling and other signals (36, 37). Interestingly, IRF8 has been implicated in maintaining HIV-1 latency in infected monocytic cell lines (34, 38, 39), and IRF4 has been implicated in negative regulation of TLR signaling (40). We therefore investigated whether various IRFs are recruited to the HIV-1 ISRE in response to LPS and GC treatment in MDMs. Using chromatin immunoprecipitation analysis, we found that IRF1, IRF2, IRF4, and IRF8 all are able to associate with the 5′ LTR and GLS containing the ISRE in HIV-infected MDMs (Fig. 5C and D). Early after treatment with LPS, the levels of IRF1 associated with this region of the viral promoter increased, whereas the levels of IRF2 and IRF4 decreased. By 24 h posttreatment with LPS, the levels of IRF4 and IRF8 associated with this region increased. It is of particular note that the levels of IRF8 recruitment increased to well above those seen in unstimulated MDMs (Fig. 5D). A similar pattern of IRF recruitment to the 5′ LTR and GLS occurred in GC-treated MDMs (Fig. 5E), suggesting that repression of HIV-1 transcription in response to LPS and GC treatment is due to enhanced IRF8 recruitment to the 5′ LTR and GLS. To confirm the central role of IRF8 in TLR4-mediated repression of HIV-1 expression in MDMs, we used shRNA to knockdown IRF8 expression in MDMs (Fig. 5F). Reducing IRF8 expression reversed TLR4-mediated HIV-1 repression in response to treatment with LPS or GC. Knockdown of IRF8 led to activation of HIV-1 expression in cells treated with a combination of PAM3CSK4 and LPS or GC, similar to that seen with treatment with PAM3CSK4 alone (Fig. 5G). In contrast, overexpression of IRF8 in MDMs led to decreased HIV-1 expression in untreated MDMs and reversed the activation of HIV-1 expression in PAM3CSK4-treated MDMs, but it had no effect on LPS-mediated repression in MDMs (Fig. 5H and I). There was a small, but significant, enhancement of HIV-1 repression in MDMs treated with a combination of PAM3CSK4 and LPS or with GC.

### Treatment with LPS or GC induces persistent low-level/latent HIV-1 infection in MDMs.

**FIG 6:**
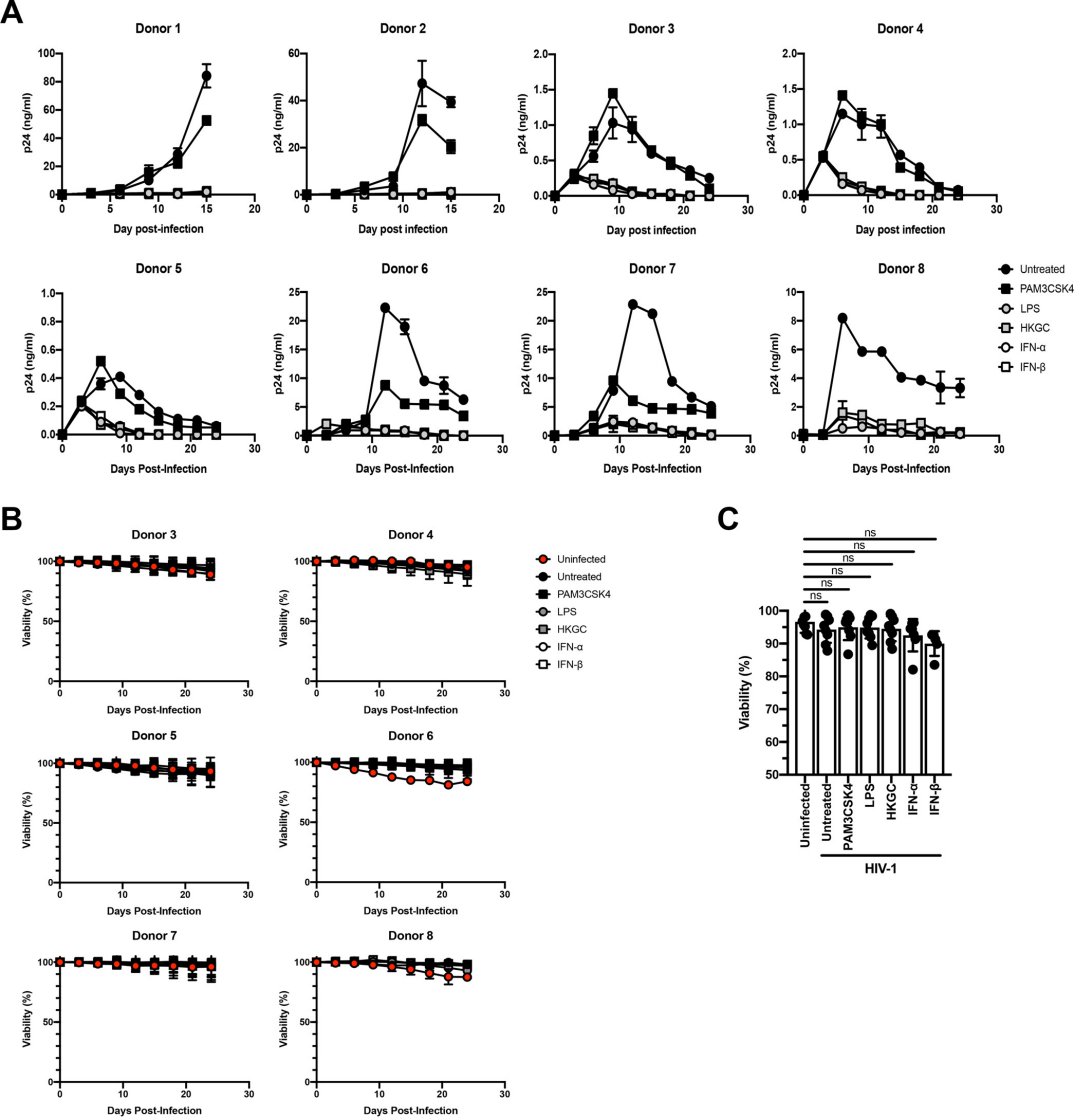
Treatment with LPS, GC, or type I IFNs induces a low-level persistent/latent infection in MDMs. MDMs were infected with replication-competent HIV-1_Ba-L_. At day 3 postinfection, MDMs from donors 1 to 5 were treated with a single dose of PAM3CSK4 (100 ng/ml), LPS (100 ng/ml), GC (MOI = 10), IFN-α (1,000 U/ml), or IFN-β (1,000 U/ml). MDMs from donors 6 and 7 were treated with PAM3CSK4, LPS, GC, or IFN-β. MDMs from donor 8 were treated with LPS, GC, or IFN-β. Cell-free supernatants were harvested every 3 days, and virus production was monitored by p24 enzyme-linked immunosorbent assay (ELISA). Data from eight independent donors, tested in triplicate (donors 1 to 5) or duplicate (donors 6 to 8), are shown. (B) Cell viability was monitored by measuring lactate dehydrogenase (LDH) in cell-free supernatants every 3 days during culture for donors 3 to 8 using a commercial LDH assay. (C) Cell viability was determined at the end of culture for donors 1 to 8 by measuring total LDH in cell lysates using a commercial LDH assay. Data from eight independent donors, tested in triplicate (donors 1 to 5) or duplicate (donors 6 to 8), are shown. *, *P* < 0.05; **, *P* < 0.01; ***, *P* < 0.001; ****, *P* < 0.0001; ns, not significant.

Recent studies in animals and human tissues demonstrate that HIV-1 can form persistent low-level or latent infections in macrophages (41–44). Our data suggest that engagement of the TLR4-TRIF-type I IFN axis in macrophages can repress virus replication and we wished to determine whether signaling through this axis could contribute to the establishment of persistent low-level or latent HIV-1 infection in macrophages. To this end, HIV-1-infected MDMs were treated a single time with the TLR2 ligand PAM3CSK4, the TLR4 ligand LPS, heat-killed GC, IFN-α, or IFN-β at day 3 postinfection. As shown in Fig. 6A, while there was a range of virus replication in the various donors, we found that treatment with a single dose of LPS, heat-killed GC, IFN-α, or IFN-β consistently led to a prominent, sustained decrease in HIV-1 replication in MDMs, whereas treatment with PAM3CSK4 led to a transient increase in HIV-1 replication followed by a slight decrease in replication. Importantly, these treatments did not significantly alter cellular viability (Fig. 6B and C). These data suggest that engagement of the TLR4-TRIF-type I IFN axis can promote low-level persistent/latent HIV-1 infection in MDMs.

**FIG 7:**
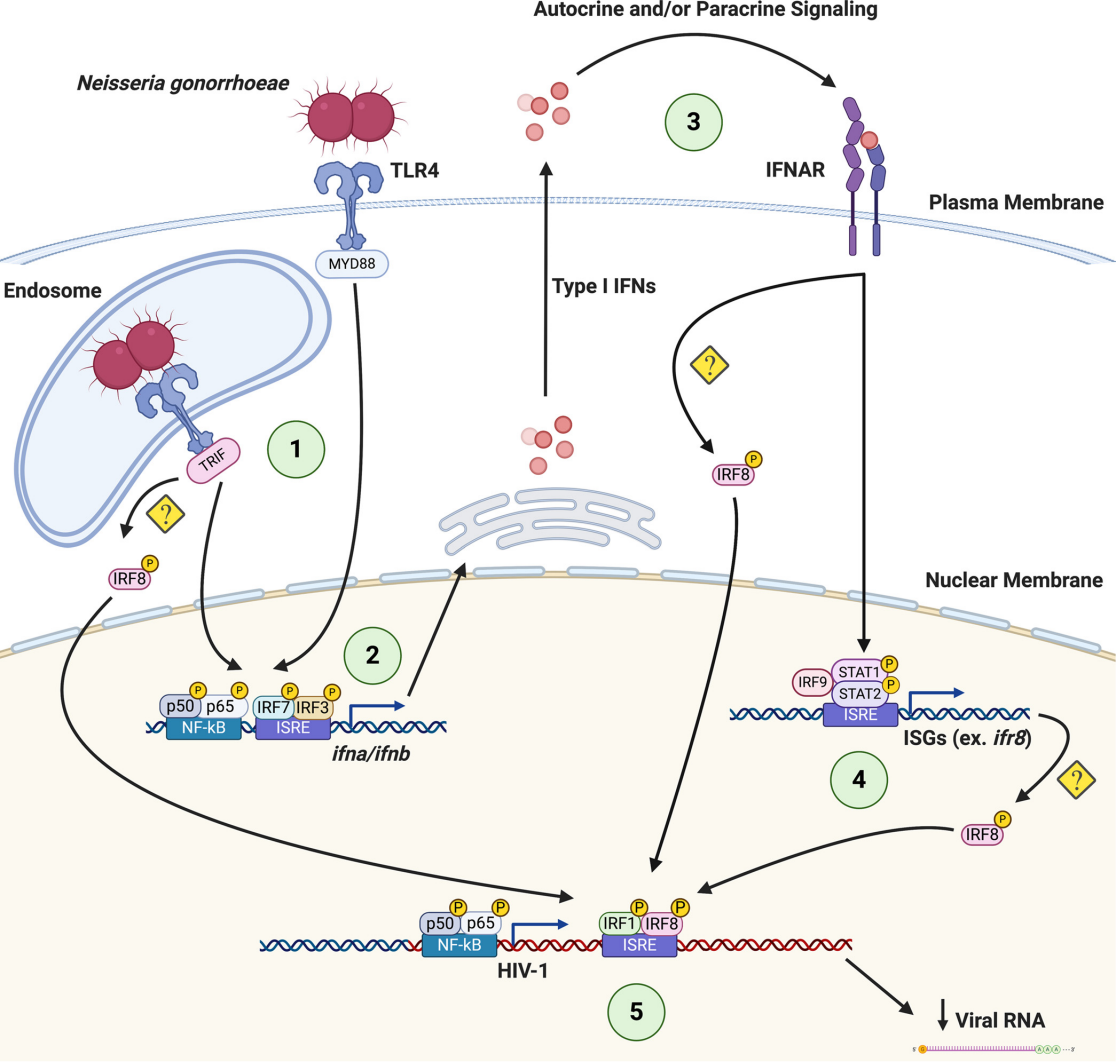
Coinfection with GC or E. coli represses HIV-1 replication by altering IRF recruitment to the HIV-1 GLS. (1) Upon engagement of TLR4 by GC (or E. coli) at the cell surface and in the endosome, signaling pathways are initiated that lead to the activation and nuclear translocation of transcription factors such as NF- κB, IRF3, and IRF7. (2) NF-κB, IRF3, and IRF7 are recruited to the IFN-α and/or IFN-β promoters to drive type I IFN expression. (3) Type I IFNs act through paracrine or autocrine signaling to drive the expression of ISGs, including IRFs (4). (5) During the late phase of the response, IRF8 is recruited to the HIV-1 GLS in a TLR4-TRIF-type I IFN-dependent manner, leading to the repression of HIV-1 transcription. Figure created with Biorender.com.

Taken together, these findings suggest that both LPS and GC activate TLR4-mediated TRIF signaling in MDMs, resulting in the production of type I IFNs. In turn, type I IFNs work in an autocrine or paracrine fashion to induce the expression of IRF8, which then binds to the ISRE present in the GLS of HIV-1 to repress viral transcription (Fig. 7).

## DISCUSSION

In these studies, we provide evidence that the interaction between commensal and pathogenic bacteria can repress HIV-1 replication in macrophages by altering the recruitment of transcription factors to the HIV-1 GLS, thereby inducing a state reminiscent of proviral latency. We further demonstrate that TLR2 ligands trigger MyD88-mediated signaling that increases virus expression via the activation of NF-κB, whereas TLR4 ligands trigger TRIF-dependent production of type I IFNs. Type I IFN signaling, in turn, is associated with the recruitment of IRF8 to the ISRE located in the GLS and a shift to low-level or latent HIV-1 infection.

A number of studies have shown that IRFs play an important role in the regulation of HIV-1 replication. There is an ISRE located downstream from the 5′ LTR in the GLS that is essential for efficient viral replication (26, 45). This ISRE is typically bound by IRF1 and/or IRF2, leading to activation of virus transcription (34, 46) through the recruitment of transcriptional coactivators, such as the histone acetyltransferase (HAT) p300/CBP (47). IRF1 and IRF2 are ubiquitously expressed in cells, although they can be upregulated by type I IFNs (36) and, in the case of IRF1, by TLR signaling (48, 49) and HIV-1 infection (45, 50), illustrating how HIV-1 can coopt the antiviral IFN response to augment its own replication. Once associated with the ISRE, IRF1 can cooperatively bind to both NF-κB at the HIV-1 LTR and the viral transactivator Tat at the HIV-1 TAR loop to augment viral transcription/elongation (34, 51). Our studies demonstrate that both IRF1 and IRF2 associate with the HIV-1 ISRE in unstimulated MDMs (Fig. 5). Upon stimulation with TLR4 ligands, IRF1 recruitment to the HIV-1 ISRE is enhanced (Fig. 5), consistent with the prevailing theory that TLR-MyD88 signaling can activate IRF1 (52). This is accompanied by a concomitant decrease of IRF2 binding. These data suggest that IRF1 binding to the ISRE as either monomers or homodimers activates HIV-1 expression, whereas IRF2 binding to the ISRE as monomers, homodimers, or heterodimers with IRF1 represses HIV-1 expression. Unfortunately, chromatin immunoprecipitation (ChIP) analysis of HIV-infected MDMs using current tools does not permit differentiating between the association of various homodimers and heterodimers with the ISRE at a population level.

We demonstrate that at late time points after TLR4 engagement, IRF8 is recruited to the GLS downstream from the 5′ LTR (Fig. 5) and that this is associated with decreased HIV-1 transcription (Fig. 1). Macrophages express high basal levels of IRF8, although its expression can be further enhanced in response to type I IFNs (36, 37) or TLR signaling (53, 54). IRF8 has been shown to bind to IRF1, in addition to other transcription factors, and to serve as either a transcriptional activator or a transcriptional inhibitor of other genes in a context-dependent manner (55–57). Previous studies have shown that IRF8 can repress HIV-1 expression (34, 38, 39). In fact, the interaction between IRF8 and IRF1 has been shown to repress HIV-1 transcription in Jurkat cells (34). This may be due to IRF8-mediated disruptions of the IRF1-Tat interaction and/or the IRF1-NF-κB interaction (51) that increase viral replication. Based on our data, we propose that changes in the IRF binding pattern to the ISRE in response to TLR signaling have profound effects on HIV-1 replication. In unstimulated HIV-infected macrophages, the ISRE is most likely occupied by IRF1/IRF2 heterodimers that allow for a low level of virus replication (Fig. 5D). Early after stimulation of TLR4 with LPS, there is a switch to IRF1 homodimers present at the ISRE that allow for high levels of virus replication due to cooperative binding between IRF1, NF-κB, and HIV-1 Tat (Fig. 5D). At late time points after TLR4 stimulation with LPS or GC, during the IFN feedback phase of the response, the ISRE is occupied by IRF1/IRF8 heterodimers (Fig. 5D). These IRF1/IRF8 heterodimers likely block the cooperative interaction(s) between IRF1, NF-κB, and Tat, thereby repressing HIV-1 replication. Although we also demonstrate that there is a transient decrease in IRF4 recruitment to the HIV-1 ISRE following treatment with LPS, the biological significance of this finding is uncertain. Prior studies have provided evidence for an LPS/TLR4-mediated repression of HIV-1 expression through the induction of type I IFNs and other mechanisms (16, 17, 58–62). Our data extend these findings and demonstrate that LPS treatment, as well as infection with the sexually transmitted pathogen GC or the gut-associated microbe E. coli, represses HIV-1 expression in MDMs through the TLR4-mediated, TRIF-dependent production of type I IFNs and the subsequent recruitment of IRF8 to the HIV-1 ISRE (Fig. 7). The exact mechanism whereby IRF8 is recruited to the HIV-1 GLS is not certain. This process may involve direct activation of IRF8 by TLR4 or the type I IFN receptor (IFNAR), or increased expression of IRF8 downstream of type I IFN signaling.

Importantly, our data suggest that the microbial environment can influence the state of HIV-1 replication and the establishment of latency in human macrophages as part of the viral reservoir in infected individuals under antiretroviral therapy (ART) regimens. Macrophages can be productively infected with HIV-1 *in vivo*, and viral replication can be modulated by copathogens through their interactions with innate immune receptors such as TLRs (18, 63). We demonstrate that productive infection of macrophages can be altered by TLR signaling in response to purified ligands and bacterial coinfection, with TLR2- and TLR5-mediated signaling activating HIV-1 and TLR3- and TLR4-mediated signaling repressing HIV-1 replication in MDMs (Fig. 1).

In addition to their role in HIV-1 production, macrophages also contribute to HIV-1 persistence *in vivo*. Although CD4^+^ memory T cells are thought to constitute the majority of the HIV-1 reservoir, several studies have demonstrated that tissue-resident macrophages in the lymph nodes (64–66), gastrointestinal tract (5, 67), genitourinary tract (2, 42, 68), liver (69–71), and lung (72–74), as well as perivascular macrophages and microglial cells in the brain (41, 75–80), can serve as tissue reservoirs for HIV-1. In simian-human immunodeficiency virus (SHIV)-infected rhesus macaques, *in vivo* viral replication was sustained by tissue macrophages after depletion of CD4^+^ T cells (81). Moreover, HIV-1 persistence in macrophages was confirmed in HIV-1-infected humanized myeloid-only mice in which viral rebound was observed in a subset of the animals following treatment interruption (43). These studies demonstrate that macrophages have the capacity to serve as bona fide HIV-1 reservoirs *in vivo*. Our findings that pathogenic and commensal bacteria, through engagement of TLRs, can influence HIV-1 replication in macrophages have potential clinical significance. For example, sexually transmitted infections (STIs) that induce robust type I interferon production, such as GC or HSV-2, may repress virus replication in genitourinary tract macrophages that harbor HIV-1 provirus and contribute to viral escape from the immune system and from ART.

The major obstacle to the eradication of HIV-1 is the presence of a persistent viral reservoir that can resurface upon discontinuation of ART. The potential contribution of HIV-1 in tissue macrophages to virus rebound with the cessation of ART is not entirely understood, but recent primate studies suggest that the functional macrophage reservoir can contribute to viral rebound upon treatment cessation (82–84). Our data demonstrate that interactions between macrophages and pathogenic or commensal microorganisms within the genitourinary and gastrointestinal tracts, such as GC and E. coli, may alter the ability of macrophages to serve as reservoirs for viral persistence in the host. Our findings are consistent with independent studies that demonstrate that repeated stimulation of M1-polarized MDMs with proinflammatory cytokines (TNF-α) and/or type II IFNs (interferon-γ) induce a state akin to HIV-1 latency (85). In addition, the oral pathogen Porphyromonas gingivalis has been shown to influence the establishment and maintenance of persistent HIV-1 infection in MDMs (86). Finally, studies have demonstrated that a subset of HIV-1-infected macrophages enter a state of viral latency characterized by altered metabolic signatures (87) and apoptotic mechanisms (88). Taken together, these studies demonstrate that coinfection, inflammatory stimuli, and metabolic alterations can influence the establishment and maintenance of the HIV-1 reservoir in macrophages. As an example, gastrointestinal macrophages constitute a major cellular reservoir for HIV-1 (5, 89–91) and are frequently exposed to microbes and microbial products either through luminal sampling (92) or microbial translocation, the latter of which is increased in HIV-positive individuals (93). Our data suggest that interactions such as those between intestinal macrophages and gut-associated microbes may have clinical significance for the establishment and maintenance of the latent HIV-1 reservoir.

Our results demonstrating that Neisseria gonorrhoeae and E. coli repress HIV-1 replication in macrophages by altering transcription factor recruitment to the HIV-1 GLS and induce a state of viral latency confirm the need for further *in vitro*, *ex vivo*, and *in vivo* studies regarding the effects of sexually transmitted pathogens and commensal microbes on HIV-1 persistence.

## MATERIALS AND METHODS

### Ethics statement.

This research has been determined to be exempt by the Institutional Review Boards of the Boston University Medical Center and University of Utah Health, since it does not meet the definition of human subject research.

### Cell isolation and culture.

Primary human CD14^+^ monocytes were isolated from the peripheral blood mononuclear cells of healthy donors using anti-CD14 magnetic beads (Miltenyi Biotec) per the manufacturer's instructions. Primary monocyte-derived macrophages (MDMs) were generated by culturing CD14^+^ monocytes in the presence of 10% human AB serum and 10% fetal bovine serum (FBS) for 6 days. Following differentiation, MDMs were cultured in RPMI 1640 supplemented with 10% FBS, 100 U/ml penicillin, 100 μg/ml streptomycin, and 0.29 mg/ml l-glutamine. The genetic sex of a subset of the donors was determined by PCR amplification of the SRY gene located on the Y chromosome. PM1 cells were cultured in RPMI 1640 supplemented with 10% FBS, 100 U/ml penicillin, 100 μg/ml streptomycin, and 0.29 mg/ml l-glutamine. 293T cells were cultured in Dulbecco’s modified Eagle’s medium (DMEM) supplemented with 10% FBS, 100 U/ml penicillin, 100 μg/ml streptomycin, and 0.29 mg/ml l-glutamine. MAGI-CCR5 cells were cultured in DMEM supplemented with 10% FBS, 100U/ml penicillin, 100 μg/ml streptomycin, 0.29 mg/ml l-glutamine, 500 μg/ml G418, 1 μg/ml puromycin, and 0.1 μg/ml hygromycin B. HEK293-TLR2^CFP^TLR1^YFP^ cells and HEK293-TLR4^CFP^/MD-2/CD14 cells were cultured in DMEM supplemented with 10% FBS, 10 μg/ml ciprofloxacin, 0.29 mg/ml l-glutamine, and 500 μg/ml G418.

### Bacterial culture.

Neisseria gonorrhoeae (GC) strain FA1090B was a generous gift from Caroline Genco. GC was cultured from a glycerol stock on GC agar plates supplemented with IsoVitalex enrichment supplement (Becton, Dickinson) in a humidified 37°C incubator with 5% CO_2_. E. coli strain DH5α was purchased from New England Biolabs and was cultured from a glycerol stock on LB agar plates at 37°C. Where indicated, bacteria were heat inactivated (heat killed) by incubation at 56°C for 2 h. Heat inactivation was monitored by culture on GC or LB agar plates as described above.

### Flow cytometry.

TLR expression on viable MDMs was assessed 8 days after isolation using antibodies against TLR2 (clone TL2.1) and TLR4 (clone HTA125) (both from eBioscience) and eFluor 450 fixable viability dye (eBioscience). MDMs were stained in plates, washed with phosphate-buffered saline (PBS), fixed using Cytofix (BD Biosciences), and then detached after incubation in PBS supplemented with 20 mM EDTA for 1 h at 4°C. Flow cytometric data were acquired using a Becton-Dickenson FACScan II or LSRFortessa instruments, and data were analyzed using FlowJo software.

### TLR ligands, interferons, and chemical inhibitors.

PAM3CSK4, FSL-1, Salmonella enterica subsp. *enterica* serovar Typhimurium flagellin (FLA-ST), poly(I·C), and E. coli K-12 LPS were obtained from InvivoGen. TLR ligands were reconstituted in endotoxin-free H_2_O. IFN-α and IFN-β were purchased from PBL InterferonSource. B18R was purchased from Abcam. BAY 11-7082, celastrol, U0126, PD95809, and SB203580 were purchased from Sigma and reconstituted in dimethyl sulfoxide (DMSO). Dynasore was purchased from Tocris Bioscience and was reconstituted in DMSO.

### Virus production.

Single-round replication-defective HIV-1 reporter viruses were generated by packaging a luciferase-expressing reporter virus, BruΔEnvLuc2, or an enhanced green fluorescent protein (GFP)-expressing reporter virus, BruΔEnvEGFP3, with the envelope glycoproteins from VSV (VSV-G). In these constructs, reporter gene expression is under the control of the 5′ LTR. Reporter virus stocks were generated by transfecting HEK293T cells using the calcium phosphate method as described previously (18). Replication competent HIV-1_Ba-L_ was generated by infection of PM1 cells as described previously (18). Virus titers were determined using MAGI-CCR5 cells, and p24^gag^ content was determined by enzyme-limited immunosorbent assay (ELISA) as described previously (18).

### Virus infections.

To assess viral replication, macrophages (2.5 × 10^5^ cells/well in 24-well plates) were incubated with VSV-G-pseudotyped HIV-luciferase reporter virus at a multiplicity of infection (MOI) of 0.1 for 4 h at 37°C. Cells were washed four to five times with PBS to remove unbound virus, and then cultured in growth medium. Following 48 h of culture, cells were treated with TLR ligands or vehicle, as indicated in the text and figure legends. After 18 h, the cells were washed twice with PBS and lysed in PBS-0.02% Triton X-100. Luciferase activity was measured using BrightGlo luciferase reagent (Promega) and an MSII luminometer.

### HIV-1 transcription.

Total cytoplasmic RNA was isolated from MDMs using the RNeasy minikit (Qiagen). RNA (100 ng) was analyzed by reverse transcription-PCR (RT-PCR) using the OneStep RT-PCR kit (Qiagen). RNA was reverse transcribed and amplified in a total volume of 50 μl containing 2.5 mM MgCl_2_, 400 μM concentrations of each deoxynucleoside triphosphate, 10 U of RNasin RNase inhibitor (Promega), 5 μCi of α-^32^P dATP, and 0.6 μM HIV-1-specific primers. RNA samples were reverse transcribed for 30 min at 50°C. After an initial denaturing step at 95°C for 15 min, cDNA products were amplified for 25 cycles, each consisting of a 30-s denaturing step at 94°C, a 45-s annealing step at 65°C, and a 1-min extension step at 72°C. The amplification concluded with a 10-minute extension step at 72°C. Samples were resolved on 5% nondenaturing polyacrylamide gels, visualized by autoradiography, and quantified in a Molecular Dynamics PhosphorImager SI using ImageQuant software. Alternatively, HIV-1 RNA was analyzed using the QuantiTect SYBR green RT-PCR kit (Qiagen) in a LightCycler 480 (Roche). The HIV-1 primers were specific for the R and U5 regions of the LTR and amplify both spliced mRNAs and genomic RNA. The HIV-1 primers were sense primer 5′-GGCTAACTAGGGAACCCACTGC-3′ and antisense primer 5′-CTGCTAGAGATTTTCCACACTGAC-3′. α-Tubulin primers were sense primer 5′-CACCCGTCTTCAGGGCTTCTTGGTTT-3′ and antisense primer 5′-CATTTCACCATCTGGTTGGCTGGCTC-3′. RNA standards corresponding to 500, 50, and 5 ng of RNA from PAM3CSK4-activated MDMs were included in each experiment to ensure that all amplifications were within the linear range of the assay.

### HIV-1 RNA stability assays.

MDMs (2 × 10^6^ cells/well in 6-well plates) were incubated with VSV-G-pseudotyped HIV-luciferase reporter virus at an MOI of 0.1 for 4 h at 37°C. Cells were washed 4 to 5 times with PBS to remove unbound virus and cultured in growth medium. Following 48 h of culture, cells were treated with TLR ligands (PAM3CSK4 or LPS at 100 ng/ml) or vehicle for 4 h. Actinomycin D (10 μg/ml) was then added to cells to block *de novo* RNA synthesis, and total cytoplasmic RNA was isolated at given times as described in the figure legends. Viral RNA was analyzed using the QuantiTect SYBR green RT-PCR kit (Qiagen) in a LightCycler 480 (Roche) with primers specific for the R and U5 regions of the LTR as described above.

### Cytokine release assays.

MDMs (2.5 × 10^5^ cells/well) were treated with PAM3CSK4 (100 ng/ml), LPS (100 ng/ml), or GC (MOI of 10) for 24 h. Cell-free culture supernatants were collected and analyzed for TNF-α (eBioscience) or IFN-β (PBL Interferon Source) release by commercially available ELISA following the manufacturer's instructions.

### Chromatin immunoprecipitation assays.

MDMs (1.2 × 10^7^) were incubated with VSV-G-pseudotyped HIV-enhanced GFP (EGFP) reporter virus at an MOI of 2 for 4 h at 37°C. Cells were washed 4 to 5 times with PBS to remove unbound virus and cultured in growth medium. Following 48 h of culture, MDMs were treated with TLR ligands for various times, as described in the text. Cells were then fixed in 1% formaldehyde for 10 min at room temperature, quenched with 125 mM glycine, and lysed in SDS lysis buffer (1% SDS, 10 mM EDTA, 50 mM Tris [pH 8.1], 1 mM phenylmethylsulfonyl fluoride [PMSF], 1 μg/ml aprotinin, and 1 μg/ml pepstatin A). Cellular lysates were sonicated using a cup horn (550 sonic dismembrator; Fisher Scientific) at a power setting of 5 with 25 20-s pulses on ice, which fragmented the chromatin to an average length of approximately 1,000 bp. Samples were diluted and immunoprecipitated with antibodies against NF-κB p65, IRF1, IRF2, IRF4, IRF8, rabbit IgG, or goat IgG (all from Santa Cruz Biotechnology). Purified DNA samples from both ChIPs and input controls were resuspended in distilled H_2_O and analyzed by semiquantitative PCR. PCR mixtures contained 10 mM Tris-HCl (pH 8.3); 50 mM KCl; 1.5 mM MgCl_2_; 100 pmol of each primer; 200 μM each dATP, dGTP, dCTP, and dTTP; 5 μCi α^32^P-dATP; and 2.5 units of AmpliTaq Gold (Applied Biosystems) in a 50-μl reaction volume. Following an initial denaturation step at 95°C for 15 min, DNA was amplified for 30 cycles, each consisting of a 30-s denaturing step at 94°C, a 45-s annealing step at 65°C, and a 1-min extension step at 72°C. Samples were electrophoresed on 5% nondenaturing polyacrylamide gels, visualized by autoradiography, and quantified using a Molecular Dynamics PhosphorImager SI using ImageQuant software. Alternatively, purified DNA from ChIPs and input controls were analyzed using the PowerUp SYBR green mastermix (Applied Biosystems) in a LightCycler 480 (Roche). The primers used to amplify specifically the HIV-1 5′ LTR and GLS were 5′-TGGAAGGGCTAATTTACTCCC-3′ (sense) and 5′-CATCTCTCTCCTTCTAGCCTC-3′ (antisense). Control amplifications of a serial dilution of purified genomic DNA from latently infected U1 cells were performed with each primer set to ensure that all amplifications were within the linear range of the reaction. To calculate the relative levels of association with the LTR, PhosphorImager data of the PCR products obtained for immunoprecipitated chromatin samples were normalized against the PCR products obtained for input DNA (% input). Values were normalized across donors and expressed as relative binding.

### LTR mutant construction.

The reported plasmid pLTR(Sp1)-luciferase was generated by PCR amplification of pNL4-3 using the sense primer 5′-CGGGGTACCCCGTGGAAGGGCTAATTTGGTCCC-3′ and the antisense primer 5′-CCGCTCGAGCGGCATCTCTCTCCTTCTAGCCTC-3′, digestion with KpnI and XhoI, and ligation into KpnI/XhoI-digested pGL3-Basic (Promega). Mutations to the NF-κB and IRF binding sites in pLTR(Sp1)-luciferase were generated using the QuikChange IIXL site-directed mutagenesis kit (Stratagene). Primers used for site-directed mutagenesis are listed in Table 1. The −158 LTR-luciferase construct was generated by deleting the LTR sequence upstream of position −158 (relative to the start site of transcription) of pNL4-3, which includes the AP-1 binding sites located in the U3 portion of the 5′ LTR, digestion of the resulting fragment with KpnI and XhoI, and ligation into KpnI/XhoI-digested pGL3-Basic (Promega).

**TABLE 1:** Primers used for PCR-based mutagenesis

Primer name	Sequence
Forward mutI NF-κB	GGACTTTCCGCTGTCTACTTTCCAGG
Reverse mutI NF-κB	CCTGGAAAGTAGACAGCGGAAAGTCC
Forward mutII NF-κB	GCTTTCTACAATCTACTTTCCGCTGG
Reverse mutII NF-κB	CCAGCGGAAAGTAGATTGTAGAAAGC
Forward mutI&II NF-κB	GCTTTCTACAATCTACTTTCCGCTGTCTACTTTCCAGG
Reverse mutI&II NF-κB	CCTGGAAAGTAGACAGCGGAAAGTAGATTGTAGAAAGC
Forward delNF-κB	GCTGACATCGAGCTTTCTACAAAGGGAGGTGTGGCCTGGGCGGG
Reverse delNF-κB	CCCGCCCAGGCCACACCTCCCTTTGTAGAAAGCTCGATGTCAGC
Forward mutISRE	GCCCGAACAGGGACTTGCCCGCGCCCGTAAAGCCAGAGGAGATC
Reverse mutISRE	GATCTCCTCTGGCTTTACGGGCGCGGGCAAGTCCCTGTTCGGGC

### shRNA knockdown of MyD88, TRIF, and IRF8.

MDMs (1.2 × 10^7^) were transfected with plasmids that encoded either a mixture of three to five shRNAs directed against MyD88, a mixture of three to five shRNAs directed against TRIF, or a mixture of three to five control shRNAs (InvivoGen) and a blasticidin resistance gene using Oligofectamine (Invitrogen) per the manufacturer’s instructions. Transfected cells were selected by culture in the presence of blasticidin for 48 h, and either used in HIV-1 replication assays or lysed for immunoblot analysis to measure MyD88 and TRIF expression using a rabbit monoclonal antibody to MyD88 (Cell Signaling Technology), a rabbit polyclonal antibody to TRIF (Cell Signaling Technology), or a mouse monoclonal antibody to β-actin (Sigma). Similarly, MDMs were transfected with plasmids that encoded either a mixture of three to five shRNAs directed against IRF8 (Sigma) or a mixture of control shRNAs (Sigma) and a puromycin resistance gene using Oligofectamine (Invitrogen) per the manufacturer’s instructions. Transfected cells were selected by culture in the presence of puromycin for 48 h and either used in HIV-1 replication assays or lysed for immunoblot analysis to measure IRF8 expression using a rabbit monoclonal antibody (Cell Signaling Technology).

### Overexpression of IRF8.

MDMs (1.2 × 10^7^) were transfected with a plasmid that encoded IRF8 (OriGene) and a neomycin resistance gene using Oligofectamine (Invitrogen) per the manufacturer’s instructions. Transfected cells were selected by culture in the presence of neomycin for 48 h and then used for HIV-1 replication assays or lysed for immunoblot analysis to measure IRF8 expression using a rabbit monoclonal antibody (Cell Signaling Technology).

### Endocytosis/phagocytosis assays.

MDMs (5 × 10^5^/well) were treated with Dynasore (80 μM) or DMSO and then incubated with pHrodo green E. coli particles (Thermo Fisher) at 1 mg/ml for 2 h at 37°C. The MDMs were then washed three times with PBS, incubated with eFluor 450 fixable viability dye (eBioscience) for 15 min at 4°C, and analyzed by flow cytometry. Flow cytometric data were acquired using a Becton-Dickenson LSRFortessa instrument, and data were analyzed using FlowJo software.

### Viability assays.

Viability of uninfected and HIV-1-infected MDMs was monitored over time (longitudinal) and at the end of culture (endpoint) using the CytoTox-One homogeneous membrane integrity assay (Promega) per the manufacturer’s instructions.

### Statistical analysis.

Comparison between experimental samples was performed with a paired two-tailed *t* test, with *P* < 0.5 denoting significant differences. Experiments were performed in triplicate using cells from a minimum of four independent donors (unless otherwise indicated) to control for interdonor variability.
